# Choline Intake Correlates with Cognitive Performance among Elder Adults in the United States

**DOI:** 10.1155/2021/2962245

**Published:** 2021-10-29

**Authors:** Lu Liu, Song Qiao, Liying Zhuang, Shanhu Xu, Linhui Chen, Qilun Lai, Wenfeng Wang

**Affiliations:** ^1^Department of Neurology, Zhejiang Hospital, Hangzhou, Zhejiang 310013, China; ^2^School of Science, Shanghai Institute of Technology, Shanghai 201418, China

## Abstract

**Objective:**

This research attempted to explore the neuroprotective effect of choline and establish evidence for future dietary recommendations and nutritional interventions to maintain a proper cognitive function among elders aged >60 years in the US.

**Method:**

This cross-sectional study retrieved data of 2,393 eligible elderly participants from the 2011-2014 National Health and Nutrition Examination Survey. Combining dietary and supplement choline intake, total choline intake was evaluated using the 24-hour dietary recall method and the dietary supplement questionnaire. Total choline intake was categorized into tertiles, which ranged at <187.60 mg/day (T1), 187.60-399.50 mg/day (T2), and >399.50 mg/day (T3). The Consortium to Establish a Registry for Alzheimer's Disease (CERAD) Word Learning subtest, Animal Fluency (AF) test, and Digit Symbol Substitution test (DSST) was used to measure cognitive function. Participants who scored the lowest 25^th^ percentile in each cognitive test were classified in the low cognitive function (LC) group. Logistic regression models were implemented to examine the association between total choline intake and the incidence of LC.

**Results:**

In the CERAD test, the risk of LC was significantly lower in T2 than T1 (OR: 0.668, 95% CI: 0.493-0.904, and *P* = 0.006) when adjusted for age, gender, BMI, alcohol consumption, and hypertension. Similarly, T2 was associated with a significantly lower risk of LC when assessed by the AF test (OR: 0.606, 95% CI: 0.580-0.724, and *P* < 0.001) and DSST (0.584, 95% CI: 0.515-0.661, and *P* < 0.001). In all three cognitive measures, the T3 of the total choline intake was not associated with cognitive function compared to T1.

**Conclusion:**

Total choline intake at 187.06-399.50 mg/day reduces the risk of LC by approximately 50% compared to intake at <187.6 mg/day. The findings of this research may be used to establish dietary recommendations and nutritional interventions to optimize the cognitive function among elders.

## 1. Introduction

Aging is the most prominent risk factor of cognitive function declines, including processing speed, attention, certain memories, language, visuospatial abilities, and executive functioning [1]. The diagnostic criteria of several cognitive disorders are defined by the Diagnostic and Statistical Manual of Mental Disorders (DSM-5) [2]. Globally, the size of the elderly population is projected to reach 2 billion by 2050, accounting for 22% of the world population [3]. The substantial growth of the elderly population has raised considerable attention regarding cognitive changes or deficits, impacting the quality of life and leading to medical and social burdens [4, 5]. Current treatment for cognitive impairment is limited. Thus, the prevention and management of age-related cognitive decline have become a global imperative.

Choline is a water-soluble micronutrient crucial for the structural integrity of cell membrane, methyl metabolism, cholinergic neurotransmission, transmembrane signaling, and lipid-cholesterol metabolism [6]. As the precursor of acetylcholine and phospholipids, choline plays a pivotal role in neurotransmission and cell signaling [7, 8]. Dysregulation of cholinergic neurotransmission is linked to several cognitive disorders, such as Alzheimer's disease (AD), the most prevalent age-related neurodegenerative disease [9]. A reduction in acetylcholine, an essential neurotransmitter for memory and learning, has been observed among patients with AD [10].

Previous researches have proposed the neuroprotective effect of choline consumption [11, 12]. In an animal study, lifelong supplementation of choline significantly improves spatial memory [13]. Maternal choline supplementation during prenatal and perinatal periods decreases the risk of cognitive disorders of the fetus in the mice model [14, 15]. In humans, choline supplementation is also associated with improved cognitive function among young and middle-aged adults [16]. However, limited human studies have investigated the effect of choline consumption on cognitive performance.

Moreover, previous studies mainly examined the therapeutic effect of choline supplementation while ignoring the dietary choline intake. Therefore, the primary purpose of this research is to examine the relationship between total choline intake, combining dietary and supplement choline intake, and cognitive performance among elders in the US.

## 2. Methods

### 2.1. Study Design

The National Health and Nutrition Examination Survey (NHANES) is a nationwide ongoing survey consisting of a household interview and a physical examination in a mobile examination center (MEC) [17]. The Center for Disease Prevention and Control has been conducting the survey on a 2-year basis since the 1960s, intending to assess the health and nutritional status of the noninstitutionalized US civilian population. The collected data was deidentified and released for public use on the NHANES official website (https://www.cdc.gov/nchs/nhanes/index.htm). The National Center for Health Statistics Ethics Review Board approved all the NHANES protocols, and informed consent was obtained from all study participants [18].

**Figure 1:**
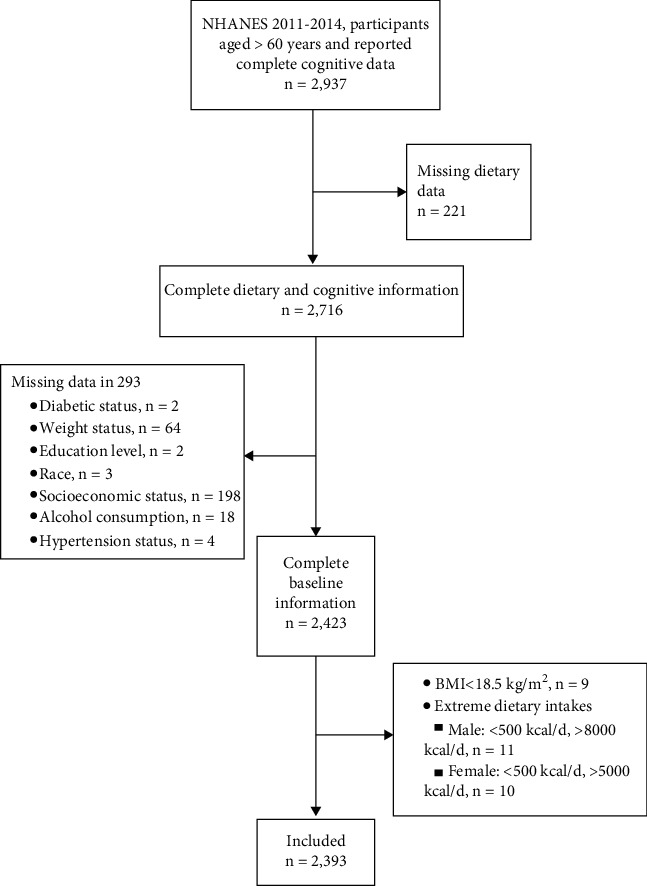
Flow chart of selecting eligible participants.

The 2011-2012 and 2013-2014 NHANES cycles evaluated the cognitive performance of the study participants and were therefore retrieved in this cross-sectional study. In this study, participants aged 60 years or older who participated in the cognitive function assessments and reported complete information were included. Participants who reported incomplete or missing data in age, gender, body mass index (BMI), race, poverty income ratio (PIR), education level, choline intake, alcohol consumption, diabetic status, hypertension status, and smoking status were excluded. Participants who indicated extreme dietary consumption (male: <500 kcal or >8000 kcal and female: <500 kcal or >5000 kcal) were excluded. Underweight people with BMI < 18.5 kg/m^2^ were also excluded. In total, 2,393 participants were eligible for the analyses in this study, as shown in Figure 1.

### 2.2. Total Choline Intake

The choline intake in this research combined the dietary and supplement intake. The total choline intake of participants with missing supplement intake is equivalent to their dietary intake. The total choline intake was categorized into the low-intake group (<25^th^ percentile), medium-intake group (25^th^-75^th^ percentile), and high-intake group (>75^th^ percentile), corresponding to choline intake at <187.60 mg/day, 187.60-399.50 mg/day, and >399.50 mg/day.

The dietary intake was collected during the MEC examination using the 24-hour dietary recall method and a USDA validated Automated Multiple-Pass Method [19]. The quantified dietary choline intake was obtained from the Dietary datasets, Dietary Interview-Total Nutrient Intakes file.

The Dietary Supplement and Prescription Medication Questionnaire (DSQ) was used to determine the supplement choline intake. The DSQ collected the total supplement intake of the participants in the past 30 days. The average daily supplement intake was calculated by averaging the total 30-day supplement intake. The Dietary Supplement Use 30 day-Total Dietary Supplements data file was accessed to obtain the total supplement choline consumption.

### 2.3. Cognitive Performance

Cognitive performance was assessed in the MEC using three cognitive function tests, the Consortium to Establish a Registry for Alzheimer's Disease (CERAD) Word Learning subset, the Animal Fluency (AF) test, and the Digit Symbol Substitution Test (DSST). Data on cognitive function was retrieved from the cognitive functioning questionnaire (CFQ).

The CERAD tested the immediate and delayed learning ability for new verbal information [20]. After visually or auditorily presenting ten words to the participants, the designated research instructed the participants to read aloud the words and recall the words immediately and after completing the AF test and DSST. Participants were asked to recall the words as many and possible. Categorical verbal fluency was assessed by the AF test [21]. Participants were asked to name as many animals as possible within 1 minute. Each named animal was counted as one point. The DSST estimated the processing speed, sustained attention, and working memory [22]. Participants were provided with a piece of paper with a key at the top and 133 boxes that adjoined numerical numbers at the bottom of the paper. The key presented the pair relationship of 9 numbers and symbols. The participants were asked to match the corresponding symbols for the 133 boxes in two minutes.

Although there was no consensus definition of low cognitive function, previous studies using the NHANES database defined participants who scored the lowest 25^th^ percentile in the cognitive tests as having low cognitive function [23, 24]. Thus, this research adopted <25^th^ percentile as the cutoff of low cognitive performance. Participants who scored CERAD < 5, AF < 13, and DSST < 34 were classified into the low cognitive function (LC) group, otherwise categorized into the normal cognitive function (NC) group.

### 2.4. Potential Covariates

This study accommodated potential covariates identified in previous studies [25, 26], containing diabetes, hypertension, weight, education level, race, social-economic status, alcohol consumption, and smoking.

Age groups were categorized on a 10-year basis, 60-69 years old, 70-79 years old, and ≥80 years old. The BMI was categorized into the normal weight group (BMI 18.5-24.9 kg/m^2^), overweight group (BMI 25-29.9 kg/m^2^), and obese group (BMI ≥ 30 kg/m^2^) based on the WHO standard [27]. Social-economic status was evaluated using the PIR, which was available in the Demographic Variables & Sample Weight (DEMO) data file. The PIR is the ratio of family income to poverty, calculated by dividing family income by the poverty guidelines. The PIR < 1 was defined as the low-income group. The Alcohol Use Questionnaire (ALQ) was used to identify alcohol consumers. Participants who answered “yes” to question ALQ101, “Had at least 12 alcohol drinks/1 yr.,” were considered alcohol consumers.

Hypertension patients were determined based on the laboratory data and Blood Pressure & Cholesterol Questionnaire (BPQ). Participants were classified as hypertension patients if their systolic pressure ≥ 140 mmHg and diastolic pressure ≥ 90 mmHg. The BPQ question 020 asked, “{Have you/Has SP} ever been told by a doctor or other health professional that {you/s/he} had hypertension, also called high blood pressure?”. Participants who answered “yes” were defined as having hypertension.

Question DIQ010 of the Diabetes Questionnaire (DIQ) asked, “The next questions are about specific medical conditions. {Other than during pregnancy, {have you/has SP}/{Have you/Has SP}} ever been told by a doctor or health professional that {you have/{he/she/SP} has} diabetes or sugar diabetes?” Participants who answered “yes” were considered diabetic. Additionally, participants with a fasting blood glucose level ≥ 7.0 mmol/L or a hemoglobin A1c ≥ 6.5% were identified as diabetic.

### 2.5. Statistical Analysis

All statistical tests were two-sided tests in this study. A *P* value less than 0.05 was considered significant. SAS 9.4 (SAS Institute, Inc., Cary, NC, USA) was used for all the statistical analyses. R 4.02 was used to generate all the graphics in this study. The distribution of variables was tested for normality by the Shapiro normality test. Nonnormally distributed continuous variables were categorized into groups. Summary statistics presented the mean and standard deviation for continuous variables (mean ± SD) while displayed frequencies and percent distributions for categorical variables (*N* %). Baseline characteristics were compared using the independent samples *t*-test, the Pearson's chi-square test (*χ*^2^), and the Fisher's exact test when appropriate. The unadjusted and multivariate logistic regression models were implemented to acquire the odds ratio (OR), 95% confidence interval (95% CI), and *P* value when investigating the association between dietary choline intake and cognitive function. The 2011-2012 and 2013-2014 NHANES cycle purposely oversampled non-Hispanic black persons, non-Hispanic non-black Asian persons, Hispanic persons, and low- and nonlow-income groups to increase the sample's representativeness in the US. Sample weights (full sample 2-year MEC examination weight) were applied to all the statistical analyses in this study. The power analysis was used to assess the statistical power (1-*β*) by the PASS 15.0 software. We found that the power values (1-*β*) of the CERAD test, the Animal Fluency (AF) test, and the Digit Symbol Substitution Test (DSST) were all 0.9, indicating that the sample size could support the multiple regression results and our findings performed well reliability.

## 3. Results

### 3.1. Study Population

The baseline characteristics were summarized and compared in Table 1. Of the included 2,393 participants, 49.02% were male, and 50.98% were female. The study population was mainly composed of non-Hispanic whites (50.02%). The PIR of most study participants was ≥1 (83.95%). More participants were obese (39.16%) than overweight (35.65%) and normal weight (25.20%). A larger percentage of alcohol consumers (69.62%) than nonconsumers (30.38%) were included in the study. Diabetic participants contributed to 23.36% of the study population. Most participants (95.45%) were not consuming dietary supplements. The total choline intake was divided into tertiles. The range of each tertile was <187.06 mg/day in T1, 187.06-399.50 mg/day in T2, and >399.50 mg/day in T3.

In all three cognition measures, the intragroup comparisons of the LC group and NC group revealed significant differences in age (*P* < 0.001), gender (*P* < 0.001), race (*P* < 0.001), an education level (*P* < 0.001), marital status (*P* < 0.001), PIR (*P* < 0.001), BMI (*P* < 0.001), alcohol consumption (*P* < 0.001), diabetes (*P* < 0.001), hypertension (*P* < 0.001), and smoking (*P* < 0.001).

In the CERAD test, of the LC group, 38.41% were 60-69 years old, 31.66% were 70-79 years old, and 29.93% were ≥80 years old; 60.73% were male, and 39.27% were female; 24.74% were normal weight (BMI 18.5 − 24.9 kg/m^2^), 41.25% were overweight (BMI 25 − 29.9 kg/m^2^), and 33.91% were obese (BMI ≥ 30 kg/m^2^); 68.17% were alcohol consumers; 65.92% had hypertension; patients with low choline intake (<187.60 mg/day) were 23.18%, medium choline intake (187.60-399.50 mg/day) were 53.81%, and high choline intake were 23.01%. In the AF test, of the LC group, 44.05% were 60-69 years old, 33.78% were 70-79 years old, and 21.82% were ≥80 years old; 48.77% were male, and 51.23% were female; 27.13% were normal weight (BMI 18.5 − 24.9 kg/m^2^), 33.97% were overweight (BMI 25 − 29.9 kg/m^2^), and 38.90% were obese (BMI ≥ 30 kg/m^2^); 63.95% were alcohol consumers; 70.40% had hypertension; patients with low choline intake (<187.60 mg/day) were 31.69%, medium choline intake (187.60-399.50 mg/day) were 46.68%, and high choline intake were 21.63%. In the DSST, of the LC group, 42.70% were 60-69 years old, 34.23% were 70-79 years old, and 23.06% were ≥80 years old; 56.22% were male, and 43.78% were female; 25.05% were normal weight (BMI 18.5 − 24.9 kg/m^2^), 35.31% were overweight (BMI 25 − 29.9 kg/m^2^), and 39.64% were obese (BMI ≥ 30 kg/m^2^); 63.06% were alcohol consumers; 71.71% had hypertension; patients with low choline intake (<187.60 mg/day) were 31.71%, medium choline intake (187.60-399.50 mg/day) were 47.93%, and high choline intake were 20.36%.

### 3.2. Choline Intake and Cognitive Performance

Three logistic regression models were implemented in this research. The crude model did not adjust for any potential covariates, while model 1 controlled for age and gender. Model 2 adjusted for age, gender, BMI, alcohol consumption, and hypertension. T1, the first tertile (<187.06 mg/day) of the total choline intake, was set as the reference group in all the logistic regression analyses. The risks of low cognitive performance in T2 (187.06-399.50 mg/day) and T3 (>399.50 mg/day) were compared with T1.

**Figure 2:**
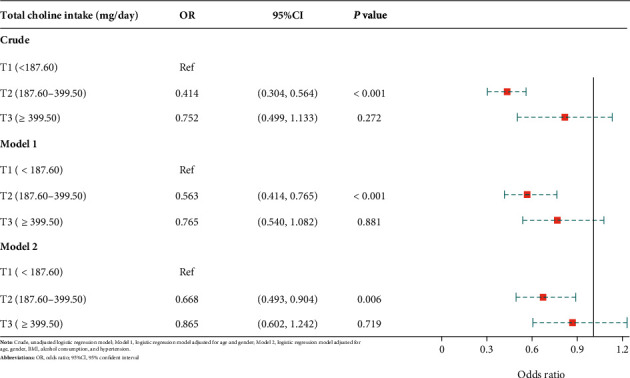
Forest plot-association between choline intake and cognitive performance assessed by the CERAD test.

In the CERAD test (Table 2 and Figure 2), T2 of the total choline intake was associated with a significantly lower incidence of declined learning ability than T1 (OR: 0.414, 95% CI: 0.304-0.564, and *P* < 0.001). The risk of low cognitive function was not statistically different in T3 compared to T1 (OR: 0.752, 95% CI:0.499-1.133, and *P* = 0.272). Model 1, adjusting for age and gender, uncovered similar results, with the risk of impaired learning ability being the lowest in T2 (OR: 0.563, 95% CI: 0.414-0.765, and *P* < 0.001). The results of model 2 (OR: 0.668, 95% CI: 0.493-0.904, and *P* = 0.006) were allied with the crude model and model 1.

**Figure 3:**
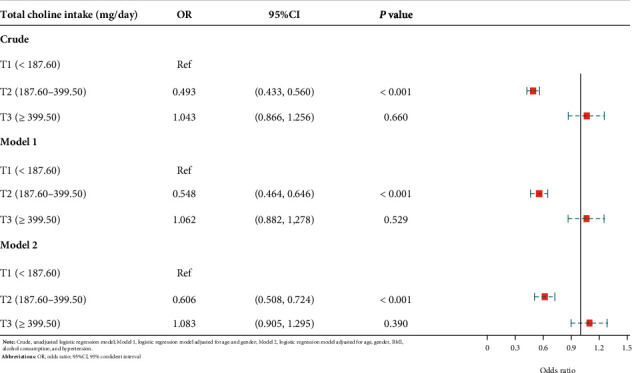
Forest plot-association between choline intake and cognitive performance assessed by the AF test.

When analyzing categorical verbal fluency using the AF test (Table 3 and Figure 3), total choline intake level at 187.06-399.50 mg/day indicated reduced odds of low categorical verbal fluency (OR: 0.493, 95% CI: 0.433-0.560, and *P* < 0.001) compared to the intake level at <187.06 mg/day. Nevertheless, no significant difference was found between the intake level > 399.50 mg/day and the intake level < 187.06 mg/day (OR:1.043, 95% CI: 0.866-1.256, and *P* = 0.660). After adjusting for covariates, the relationship remained in model 1, revealing significantly lower odds of impaired categorical verbal fluency in T2 when compared to T1 (OR: 0.548, 95% CI: 0.464-0.646, and *P* < 0.001). When further controlling for BMI, alcohol consumption, and hypertension in model 2, T2 still showed a decreased occurrence of low categorical verbal fluency (OR: 0.606, 95% CI: 0.580-0.724, and *P* < 0.001).

**Figure 4:**
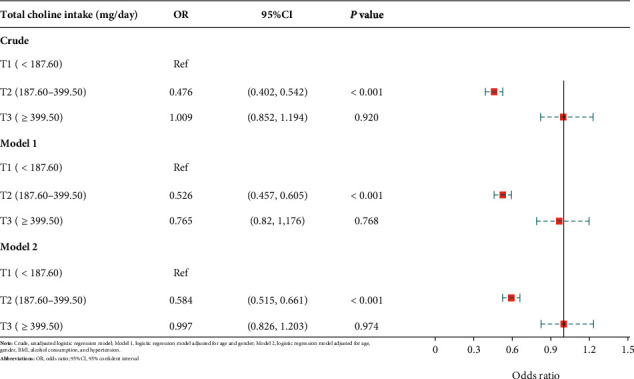
Forest plot-association between choline intake and cognitive performance assessed by the DSST.

As demonstrated in Table 4 and Figure 4, the risk of declined processing speed, sustained attention, and working memory in the T2 of total choline intake was significantly lower than that of the T1 when evaluating the cognitive performance by the DSST (OR: 0.476, 95% CI: 0.402-0.542, and *P* < 0.001). There was no significant difference when comparing the risk of low cognitive performance in T3 to the reference (OR: 1.009, 95% CI: 0.852-1.194, and *P* = 0.920). A similar pattern was observed when adjusting for age and gender, with the risk of low cognitive performance being the lowest in T2 (OR: 0.525, 95% CI: 0.457-0.605, and *P* < 0.001), while no significant difference between T1 and T3 (OR:0.765, 95% CI: 0.802-1.176, and *P* = 0.766). After further controlling for BMI, alcohol consumption, and hypertension, the results were consistent with the crude model and model 1 (OR: 0.584, 95% CI: 0.515-0.661, and *P* < 0.001).

## 4. Discussion

This research analyzed the combined data of the 2011-2012 and 2013-2014 NHANES datasets. Compared to choline intake at <187.6 mg/day, intake at 187.6-399.5 mg/day decreases the risk of low cognitive performance in learning ability, categorical verbal fluency, working memory, processing speed, and sustained attention. The risk of low cognitive performance reduces approximately 40% in all three cognition measures when the participants consume 187.6-399.5 mg choline per day.

Although choline intake at 187.6-399.5 mg/day indicates a beneficial effect, no change in the cognitive performance was observed when choline intake reached greater than 399.5 mg/day. The relationship assembles a “U” shape risk as choline consumption increases, implying there might be an optimal level of choline consumption to attenuate age-related cognitive declines. The good dietary choline sources are mainly from animal products, such as beef liver (3 oz provides 356 mg) and egg (1 large egg provides 147 mg) [28]. Although it remains controversial, consumption of red meat may increase the risk of AD by elevating brain iron levels [29]. As dietary choline intake increases, the consumption of animal products also rises, which may explain the unchanged risk of low cognitive performance when the choline consumption achieves >399.5 mg/day in our study.

Currently, the tolerable upper limit of choline intake is 3.5 g/day. The recommended daily allowance (RDA) and estimated average requirement (EAR) of choline intake have not been established due to insufficient evidence. The adequate intake (AI) is developed when insufficient data is available. The AI of choline for adults is 425 mg/day for women and 550 mg/day for men [8]. Nevertheless, our findings suggest that the optimal level of choline intake to prevent the progression of cognitive decline is lower than the AI. Therefore, results of our study may be used for the evidence-based dietary recommendation for the elders, aiming to achieve a proper cognitive function.

The underlying mechanism of choline influencing cognition has been investigated in some previous studies. Patients with AD display a reduced level of acetylcholine [30], which activates microglia in the hippocampus and leads to cascade reaction of brain inflammation and neuronal death [13]. The epigenetic mechanism has also been proposed, demonstrating the essential role of choline as an epigenetic modifier [31]. As a critical methyl donor that involves DNA and histone methylation, choline may alter brain function by modulating neuronal gene expression, such as influencing the availability of S-adenosylmethionine. Thus, identifying effective nutritional strategies is a cost-effective approach to optimize cognitive function among elders.

The Framingham Heart Study Offspring Study, a cohort study conducted in the US, measured the total choline intake using the Harvard FFQ and examined its relationship to cognitive impairment among participants aged 36-83 years [32]. Cognitive function was evaluated by a neuropsychological test battery, consisting of verbal memory, visual memory, verbal learning, and executive function assessments, and the brain MRI, evaluating the white-matter hyperintensity. The study revealed that choline consumption at midlife acted as a neuroprotective agent by decreasing the white-matter hyperintensity volume. The effectiveness of choline intake in the Framingham Heart Study was allied with the findings of our study. However, the Framingham Heart Study investigated the effectiveness of choline intake at midlife, while the consumption at elder life was not evaluated.

Low plasma choline concentration was also found to associate with poor cognitive performance. Nurk et al. conducted a cross-sectional study, attempting to research the relationship between plasma-free choline and cognitive performance [33]. The cognitive tests were administered to 2,195 participants aged 70-74 years. Nonfasting blood was collected to obtain the plasma choline concentration. High plasma choline concentration was associated with better sensorimotor speed, perceptual speed, and executive function. Many reports have shown that supplementing the maternal (during gestation and lactation) diet with additional choline benefits cognition [34–36]. A recent study showed that Chinese ischemic stroke patients with higher choline and betaine levels had a lower risk of cognitive impairment, using data derived from CATIS (China Antihypertensive Trial in Acute Ischemic Stroke) [37]. The effectiveness of choline intake was allied with the findings of our study.

In the elderly population, the neuroprotective effect on the age-related cognitive decline has not been ascertained due to limited research. The findings of our study provide evidence to establish the protective effect of choline intake on cognitive performance. Nevertheless, there are several limitations of this research. The cross-sectional design of this research cannot establish a causal relationship between choline intake and cognitive performance. Furthermore, this retrospective study could not control all behavioral, medical, and environmental factors influencing cognitive functioning. The plasma choline level related to the baseline plasma choline status was not available for consideration in this study. Thus, the therapeutic effect of choline may not be fully uncovered in this research. Finally, it is well known that food intake changes daily, and as such, a 24 h recall is not necessarily representative of usual food intake [38]. However, we have excluded underweight participants and participants with extreme intakes to reduce the incidence of potential choline deficiency or extremely low choline intake.

Future research may administer nutrition interventions considering the baseline choline status to explore the ideal choline intake to maintain healthy physiological functions and proper cognitive performance.

## 5. Conclusion

Compared to <187.6 mg/day, the total choline intake at 187.06-399.50 mg/day illustrates a protective effect on cognitive function, including learning ability, categorical verbal fluency, processing speed, sustained attention, and working memory. The results of this study may provide evidence to support and establish dietary choline recommendations for the elders and identify ideal dietary interventions to prevent age-related cognitive decline and maintain a proper cognitive function.

## Figures and Tables

**Table 1:** Baseline characteristics of the 2,393 study participants, 2011-2014 NHANES.

Variables	Total (*n* = 2,393)	CERAD		*P*	AF		*P*	DSST		*P*
		LC (*n* = 578)	NC (*n* = 1,815)		LC (*n* = 527)	NC (*n* = 1,866)		LC (*n* = 555)	NC (*n* = 1,838)	
Age, *n* (%)				<0.001			<0.001			<0.001
60-69	1,322 (55.24)	222 (38.41)	1,100 (60.61)		237 (44.05)	1,098 (58.22)		237 (42.70)	1,085 (59.03)	
70-79	703 (29.38)	183 (31.66)	520 (28.65)		178 (33.78)	531 (28.15)		190 (34.23)	513 (27.91)	
≥80	368 (15.38)	173 (29.93)	195 (10.74)		115 (21.82)	257 (13.63)		128 (23.06)	240 (13.06)	
Gender, *n* (%)				<0.001			<0.001			<0.001
Male	1,173 (49.02)	351 (60.73)	822 (45.29)		257 (48.77)	924 (48.99)		312 (56.22)	861 (46.84)	
Female	1,220 (50.98)	227 (39.27)	993 (54.71)		270 (51.23)	962 (51.01)		243 (43.78)	977 (53.16)	
Race, *n* (%)				<0.001			<0.001			<0.001
Mexican American	206 (8.61)	57 (9.86)	149 (8.21)		37 (7.02)	169 (8.96)		75 (13.51)	131 (7.13)	
Other Hispanic	238 (9.95)	64 (11.07)	174 (9.59)		68 (12.90)	170 (9.01)		102 (18.38)	136 (7.40)	
Non-Hispanic white	1,197 (50.02)	286 (49.48)	911 (50.19)		185 (35.10)	1,025 (54.35)		169 (30.45)	1,028 (55.93)	
Non-Hispanic black	560 (23.40)	145 (25.09)	415 (22.87)		185 (35.10)	379 (20.10)		190 (34.23)	370 (20.13)	
Other	192 (8.02)	26 (4.50)	166 (9.15)		52 (9.87)	143 (7.58)		19 (3.42)	173 (9.41)	
Education level, *n* (%)				<0.001			<0.001			<0.001
Less than 9th grade	244 (10.20)	109 (18.86)	135 (7.44)		102 (19.35)	142 (7.53)		188 (33.87)	56 (3.05)	
9-11th grade	319 (13.33)	89 (15.40)	230 (12.67)		100 (18.98)	222 (11.77)		118 (21.26)	201 (10.94)	
High school graduate	557 (23.28)	148 (25.61)	409 (22.53)		148 (28.08)	412 (21.85)		128 (23.06)	429 (23.34)	
Some college	704 (29.42)	128 (22.15)	576 (31.74)		116 (22.01)	593 (31.44)		87 (15.68)	617 (33.57)	
College graduate	569 (23.78)	104 (17.99)	465 (25.62)		61 (11.57)	517 (27.41)		34 (6.13)	535 (29.11)	
Marital status, *n* (%)				<0.001			<0.001			<0.001
Married	1,333 (55.70)	308 (53.29)	1,025 (56.47)		267 (50.66)	1,074 (56.95)		258 (46.49)	1,075 (58.49)	
Widowed	440 (18.39)	138 (23.88)	302 (16.64)		133 (25.24)	312 (16.54)		147 (26.49)	293 (15.94)	
Divorced	358 (14.96)	66 (11.42)	292 (16.09)		72 (13.66)	291 (15.43)		72 (12.97)	286 (15.56)	
Separated	65 (2.72)	20 (3.46)	45 (2.48)		21 (3.98)	45 (2.39)		33 (5.95)	32 (1.74)	
Never married	132 (5.52)	27 (4.67)	105 (5.79)		24 (4.55)	109 (5.78)		29 (5.23)	103 (5.60)	
Living with partner	65 (2.72)	19 (3.29)	46 (2.53)		10 (1.90)	55 (2.92)		16 (2.88)	49 (2.67)	
PIR, *n* (%)				<0.001			<0.001			<0.001
<1	384 (16.05)	109 (18.86)	275 (15.15)		135 (25.62)	256 (13.57)		171 (30.81)	213 (11.59)	
≥ 1	2,009 (83.95)	469 (81.14)	1,540 (84.85)		392 (74.38)	1,630 (86.43)		384 (69.19)	1,625 (88.41)	
BMI, *n* (%)				<0.001			<0.001			<0.001
18.5 to <25	603 (25.20)	143 (24.74)	460 (25.34)		143 (27.13)	460 (24.39)		139 (25.05)	464 (25.24)	
25 to <30	853 (35.65)	239 (41.35)	614 (33.83)		179 (33.97)	674 (35.74)		196 (35.32)	657 (35.75)	
≥ 30	937 (39.16)	196 (33.91)	741 (40.83)		205 (38.90)	732 (38.81)		220 (39.64)	717 (39.01)	
Alcohol consumption, *n* (%)				<0.001			<0.001			<0.001
Yes	1,666 (69.62)	394 (68.17)	1,272 (70.08)		337 (63.95)	1,343 (71.21)		350 (63.06)	1,316 (71.60)	
No	727 (30.38)	184 (31.83)	543 (29.92)		190 (36.05)	543 (28.79)		205 (36.94)	522 (28.40)	
Diabetes, *n* (%)				<0.001			<0.001			<0.001
Yes	559 (23.36)	149 (25.78)	410 (22.59)		158 (29.98)	401 (21.26)		180 (32.43)	379 (20.62)	
No	1,721 (71.92)	400 (69.20)	1,321 (72.78)		347 (65.84)	1,394 (73.91)		352 (63.42)	1,369 (74.48)	
Borderline	113 (4.72)	29 (5.02)	84 (4.63)		22 (4.17)	91 (4.83)		23 (4.14)	90 (4.90)	
Hypertension, *n* (%)				<0.001			<0.001			<0.001
Yes	1,502 (62.77)	381 (65.92)	1,121 (61.76)		371 (70.40)	1,139 (60.39)		398 (71.71)	1,104 (60.07)	
No	891 (37.23)	197 (34.08)	694 (38.24)		156 (29.60)	747 (39.61)		157 (28.29)	734 (39.93)	
Smoking, *n* (%)				<0.001			<0.001			<0.001
Yes	1,230 (51.40)	291 (50.35)	939 (51.74)		276 (52.37)	966 (51.22)		300 (53.10)	936 (50.92)	
No	1,163 (48.60)	287 (49.65)	876 (48.26)		251 (47.63)	920 (48.78)		265 (46.90)	902 (49.08)	
Supplement use, *n* (%)				<0.001			<0.001			<0.001
Yes	109 (4.55)	22 (3.81)	87 (4.79)		13 (2.47)	101 (5.36)		11 (1.98)	98 (5.33)	
No	2,284 (95.45)	556 (96.19)	1,728 (95.21)		514 (97.53)	1,785 (94.64)		544 (98.02)	1,740 (94.67)	
Choline intake tertiles (mg)				<0.001			<0.001			<0.001
<187.60	598 (24.99)	134 (23.18)	464 (25.56)		167 (31.69)	436 (23.12)		176 (31.71)	422 (22.96)	
187.60 to <399.50	1,197 (50.02)	311 (53.81)	886 (48.82)		246 (46.68)	961 (50.95)		266 (47.93)	931 (50.65)	
≥ 399.50	598 (24.99)	133 (23.01)	465 (25.62)		114 (21.63)	489 (25.93)		113 (20.36)	485 (26.39)	

CERAD: the Consortium to Establish a Registry for Alzheimer's Disease; AF: Animal Fluency test; DSST: the Digit Symbol Substitution Test; LC: low cognitive function group; NC: normal cognitive function group; PIR: poverty income ratio; BMI: body mass index.

**Table 2:** The association between choline intake and cognitive performance assessed by the CERAD test.

Variables	Crude		Model 1		Model 2	
	OR (95% CI)	*P*	OR (95% CI)	*P*	OR (95% CI)	*P*
Total choline intake (mg/day)						
T1 (<187.06)	Ref		Ref		Ref	
T2 (187.06-399.50)	0.414 (0.304-0.564)	<0.001	0.563 (0.414-0.765)	<0.001	0.668 (0.493-0.904)	0.006
T3 (>399.50)	0.752 (0.499-1.133)	0.272	0.765 (0.540-1.082)	0.881	0.865 (0.602-1.242)	0.719

Crude: unadjusted logistic regression model; model 1: logistic regression model adjusted for age and gender; model 2: logistic regression model adjusted for age, gender, BMI, alcohol consumption, and hypertension; OR: odds ratio; CI: confidence interval.

**Table 3:** The association between choline intake and cognitive performance assessed by the AF test.

Variables	Crude		Model 1		Model 2	
	OR (95% CI)	*P*	OR (95% CI)	*P*	OR (95% CI)	*P*
Total choline intake (mg/day)						
T1 (<187.06)	Ref		Ref		Ref	
T2 (187.06-399.50)	0.493 (0.433-0.560)	<0.001	0.548 (0.464-0.646)	<0.001	0.606 (0.508-0.724)	<0.001
T3 (>399.50)	1.043 (0.866-1.256)	0.660	1.062 (0.882-1.276)	0.529	1.083 (0.905-1.295)	0.390

Crude: unadjusted logistic regression model; model 1: logistic regression model adjusted for age and gender; model 2: logistic regression model adjusted for age, gender, BMI, alcohol consumption, and hypertension; OR: odds ratio; CI: confidence interval.

**Table 4:** The association between choline intake and cognitive performance assessed by the DSST.

Variables	Crude		Model 1		Model 2	
	OR (95% CI)	*P*	OR (95% CI)	*P*	OR (95% CI)	*P*
Total choline intake (mg/day)						
T1 (<187.06)	Ref		Ref		Ref	
T2 (187.06-399.50)	0.476 (0.402-0.542)	<0.001	0.526 (0.457-0.605)	<0.001	0.584 (0.515-0.661)	<0.001
T3 (>399.50)	1.009 (0.852-1.194)	0.920	0.765 (0.802-1.176)	0.786	0.997 (0.826-1.203)	0.974

Crude: unadjusted logistic regression model; model 1: logistic regression model adjusted for age and gender; model 2: logistic regression model adjusted for age, gender, BMI, alcohol consumption, and hypertension; OR: odds ratio; CI: confidence interval.

## Data Availability

The data utilized to support the findings are available from the corresponding authors upon request.

## Abbreviations

AD: Alzheimer's disease
NHANES: National Health and Nutrition Examination Survey
MEC: Mobile examination center
DSQ: Dietary Supplement and Prescription Medication Questionnaire
CERAD: Consortium to Establish a Registry for Alzheimer's Disease
DSST: Digit Symbol Substitution test
CFQ: Cognitive functioning questionnaire
LC: Low cognitive function
NC: Normal cognitive function
BMI: Body mass index
PIR: Poverty income ratio
OR: Odds ratio
95% CI: 95% confidence interval
AI: Adequate intake.
